# Advances in Microfluidic Paper-Based Analytical Devices for Food and Water Analysis

**DOI:** 10.3390/mi7050086

**Published:** 2016-05-09

**Authors:** Lori Shayne Alamo Busa, Saeed Mohammadi, Masatoshi Maeki, Akihiko Ishida, Hirofumi Tani, Manabu Tokeshi

**Affiliations:** 1 Graduate School of Chemical Sciences and Engineering, Hokkaido University, Kita 13 Nishi 8, Kita-ku, Sapporo 060-8628, Japan; lorishayne_busa@eis.hokudai.ac.jp (L.S.A.B.); drsaeedmoh@ec.hokudai.ac.jp (S.M.); 2 Physical Sciences Department, Nueva Vizcaya State University, Bayombong, Nueva Vizcaya 3700, Philippines; 3 Division of Applied Chemistry, Faculty of Engineering, Hokkaido University, Kita 13 Nishi 8, Kita-ku, Sapporo 060-8628, Japan; m.maeki@eng.hokudai.ac.jp (M.M.); ishida-a@eng.hokudai.ac.jp (A.I.); tani@eng.hokudai.ac.jp (H.T.); 4 ImPACT Research Center for Advanced Nanobiodevices, Nagoya University, Furo-cho, Chikusa-ku, Nagoya 464-8603, Japan; 5 Innovative Research Center for Preventive Medical Engineering, Nagoya University, Furo-cho, Chikusa-ku, Nagoya 464-8601, Japan; 6 Institute of Innovation for Future Society, Nagoya University, Furo-cho, Chikusa-ku, Nagoya 464-8601, Japan

**Keywords:** μPADs, food analysis, water analysis, point-of-need

## Abstract

Food and water contamination cause safety and health concerns to both animals and humans. Conventional methods for monitoring food and water contamination are often laborious and require highly skilled technicians to perform the measurements, making the quest for developing simpler and cost-effective techniques for rapid monitoring incessant. Since the pioneering works of Whitesides’ group from 2007, interest has been strong in the development and application of microfluidic paper-based analytical devices (μPADs) for food and water analysis, which allow easy, rapid and cost-effective point-of-need screening of the targets. This paper reviews recently reported μPADs that incorporate different detection methods such as colorimetric, electrochemical, fluorescence, chemiluminescence, and electrochemiluminescence techniques for food and water analysis.

## 1. Introduction

Ensuring the safety and quality of food is an incessant concern. Hamburg’s editorial in *Science* entitled “Advancing regulatory science” [1] states the relevance of this matter, and indeed, one of the key points of food analysis is to ensure food safety [2]. In order to meet this goal, there is a constant search for new and more practical methods for food monitoring. Food is after all the source of nutrition and energy of every human. Similarly, water safety and quality is of great importance. With water being the major constituent of the human body, it is natural that enough water must be consumed to regulate bodily functions [3]. However, failure to warrant the safety and quality of food and water brings risks that often lead to illnesses and sometimes fatalities.

The safety of food and water is often affected by several factors, including the presence of pathogens, pesticides and herbicides, metals and other toxic materials generally borne to the food and water through agricultural and industrial processes. Another influencing factor is the amount of food additives used to provide food preservation, coloring and sweetening [4]. Such food additives have to be controlled due to the potential risks that these substances pose to human health. Some have even become prohibited due to their toxicity such as furylfuramide (AF-2), which was used as food preservative in Japan from 1965 or earlier; it was later banned due to its carcinogenicity in experimental animals [5].

This review discusses the recent progress in microfluidic paper-based analytical device (μPAD) technology for food and water safety monitoring, specifically μPAD applications to the detection of different target compounds and pathogens that are either borne naturally to food and water, or caused by unmonitored industrial and agricultural processing and waste contamination to both. Lateral-flow immunoassays (also known as immunochromatographic assays) are excluded as they have been reviewed elsewhere [6,7]. This review also covers the types of paper substrates that have been utilized in the μPAD fabrication and the detection methods that were incorporated into the μPAD for specific target detection for food and water analysis.

## 2. Paper in Microfluidics

Microfluidics as defined by Whitesides [8] in his article published in *Nature* in 2006 is the science and technology of systems that process and manipulate small amounts of fluid up to 10^−9^ to 10^−18^ L using fluidic channels with dimensions ranging from tens to hundreds of micrometers. Microfluidics has undergone rapid growth with notable impacts to the analytical chemistry community due to a number of capabilities including its ability to utilize small amounts of samples and reagents and to perform separation and detection with high resolution and sensitivity, at low cost and rapidly [9]. Some of the early reports on microfluidic fabrication involved the use of glass [10,11], silicon [12,13], and polymers such as poly(dimethylsiloxane) (PDMS) [14,15] as substrates. Though these microfluidic devices miniaturize the conventional methods for specific target separation and detection, they have some drawbacks such as the expense of the substrate materials, and the need for power supply and fluid transport instruments.

Paper on the other hand is a very promising substrate material for microfluidic device fabrication for a number of reasons. The properties of paper and the many advantages that it provides as a low-cost platform for diagnostics have been well-discussed [16,17,18]: It is easily printed, coated and impregnated; its cellulose composition is particularly compatible with proteins and biomolecules; it is environment-compatible as it is easily disposed of by incineration; and it is accessible almost everywhere. With paper as its main substrate, the cellulose membrane network of the microfluidic paper-based analytical devices (μPADs) provide instrument-free liquid transport by capillary action, a high surface area to volume ratio that enhances detection limits for colorimetric assays, and the ability to store chemical components in their active form within the paper fiber network [19]. Although μPADs lack the high resolution and sensitivity that the silicon, glass or plastic-based devices offer, the application of μPADs is highly suitable to point-of-need monitoring that requires inexpensive analysis for constant testing especially in less industrialized countries where complex instrumentation and analytical laboratories and experts are limited. Hence, μPADs have emerged as an attractive alternative to highly sophisticated instrumentation in analytical research applications particularly in food and water monitoring and safety.

**Figure 1:**
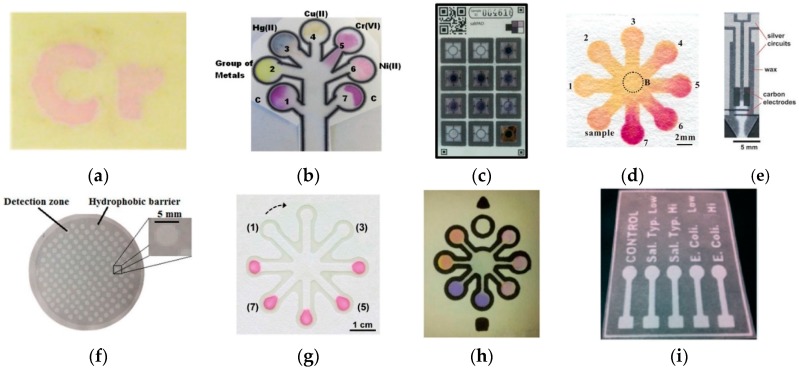
Examples of μPADs fabricated using different methods and paper substrates: (**a**) Wax patterning, WCP1. Reprinted with permission from reference [28]. Copyright 2015 American Chemical Society. (**b**) Wax printing, WP1. Reprinted with permission from reference [31]. Copyright 2011 American Chemical Society. (**c**) Wax printing, AP319. Reprinted with permission from reference [39]. Copyright 2015 American Chemical Society. (**d**) Alkylsilane self-assembling and UV/O_3_-patterning, WFP1. Reprinted with permission from reference [52]. Copyright 2013 American Chemical Society. (**e**) Wax printing with screen-printed electrodes, WCP1. Reprinted with permission from reference [38]. Copyright 2010 The Royal Society of Chemistry. (**f**) Polymer screen printing, WFP4. Reprinted with permission from reference [42]. Copyright 2016 The Royal Society of Chemistry. (**g**) Contact stamping, JPFP40. Reprinted with permission from reference [44]. Copyright 2015 The Royal Society of Chemistry. (**h**) Contact stamping, WFP1. Reprinted with permission from reference [45]. Copyright 2014 American Chemical Society. (**i**) Photolithography, CP. Reprinted with permission from reference [46]. Copyright 2013 The Royal Society of Chemistry. WFP1, Whatman No. 1 filter paper; WCP1, Whatman No. chromatography paper; WP1, Whatman No. 1 paper; AP310, Ahlstrom 319 paper; WFP4, Whatman No. 4 filter paper; JPFP40, JProLab JP 40 filter paper; CP, chromatography paper.

To date, much analytical research has focused on the development and application of μPADs for food and water safety and quality monitoring; including fabrication procedures of the μPADs and suitable methods of detection for qualitative or quantitative interpretation of measurements. Fabrication usually entails the selection of a type of paper substrate before subjecting it to fabrication techniques such as cutting [20,21,22,23,24,25], inkjet printing [26,27], wax patterning [28,29], wax pencil drawing [30], wax printing [31,32,33,34,35,36,37,38,39,40], screen printing [29,41,42], contact stamping [43,44,45], and photolithography [46,47,48]. Examples of μPADs fabricated using various methods and paper substrates are shown in Figure 1. Among the various cellulose-based paper substrates that have been used, Whatman chromatography paper grade 1 was the first type to be utilized in 2007 [17] and it has been subsequently used in many reported μPAD fabrication and detection methods [28,29,33,37,38,47,49,50]. Whatman filter paper grade 1, on the other hand, has been the most commonly used paper substrate for μPAD fabrication in food and water analysis [25,30,32,34,35,36,41,45,51,52,53,54]. Paper substrates that have been similarly utilized include Whatman chromatography paper 3 MM Chr [20,21], Whatman filter paper grade 4 [42,55], Whatman RC60 regenerated cellulose membrane filter [56], Millipore MCE membrane filter [57], Canson paper [58], Fisherbrand P5 filter paper [59], JProLab JP 40 filter paper [44], Advantec 51B chromatography paper [48], and Ahlstrom 319 paper [39]. Although comparing the capabilities of each paper substrate is inappropriate when different fabrication methods and detection methods are employed among the studies, some comparisons of substrates have been made. Liu *et al.* [20], for instance, investigated paper substrates including nitrocellulose membrane, filter paper, quantitative filter paper, qualified filter paper and Whatman 3 mm chromatography paper for the μPAD chemiluminescence (CL) detection of dichlorvos (DDV) in vegetables. With the filter paper, quantitative filter paper and qualified filter paper, a high CL signal of the blank sample and poor repeatability for sample detection were observed due to the non-uniform thickness of the substrates (from 10 to 250 μm) affecting the optical path length, scattering, assay sensitivity, and volume of fluid required for an assay. However, Whatman 3 mm chromatography paper, which has high quality, purity and consistency, provided good repeatability.

## 3. Applications to Food and Water Contamination

### 3.1. Detection of Foodborne and Waterborne Pathogens

**Figure 2:**
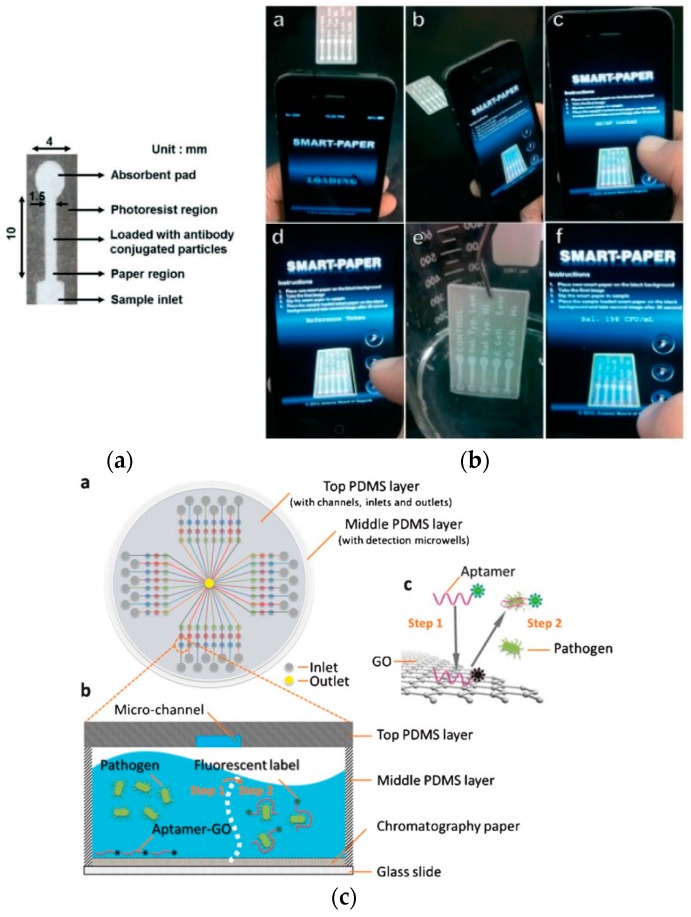
Detection methods for pathogens. (**a**) An image of a single-channel μPAD and (**b**) the smartphone application for *Salmonella* detection on a multi-channel μPAD. Reprinted with permission from reference [46]. Copyright 2013 The Royal Society of Chemistry. (**c**) Schematic layout of the PDMS/paper hybrid μPAD system and illustration of the one-step multiplexed FL detection principle on the μPAD during aptamer adsorption (Step 1) and liberation (Step 2) from the GO surface and the restoration of the FL for detection in the presence of the target pathogen. Reprinted with permission from reference [60]. Copyright 2013 The Royal Society of Chemistry.

Paper-based approaches for food safety monitoring are attractive because simple, low-cost, and on-site detection of foodborne contaminants is achievable and they are also applicable as preventive measures. μPADs developed for pathogen detection in food have relied primarily on enzymatic assay-based optical methods where results are either confirmed visually by the naked eye or digitally converted and measured using image analysis software. Two of the most commonly used programs are ImageJ and Adobe Photoshop where RGB (red-green-blue) image intensities are measured relative to the image pixels or are first converted into CMYK (cyan-magenta-yellow-key) scale before intensity measurement. In a study reported by Jokerst *et al.* [32], a μPAD was developed for the microspot assay of *Escherichia coli* (*E. coli*) O157:H7, *Listeria monocytogenes* (*L. monocytogenes*) and *Salmonella* Typhimurium in ready-to-eat meat samples. The pathogens were collected from foods by a swab sampling technique and then cultured in media before adding to a chromogen-impregnated paper-based well device. A color change is observed indicating the presence of an enzyme associated with the pathogen of interest and detection is achieved. Although the detection limits determined for each of the live bacterial assays after ImageJ analysis were high (10^6^ colony-forming unit (CFU) mL^−1^ for *E. coli*, 10^4^ CFU mL^−1^ for *Salmonella* Typhimurium, and 10^8^ CFU mL^−1^ for *L. monocytogenes*), the developed μPAD was capable of detecting pathogenic bacteria in ready-to-eat meat (bologna) at a concentration of as low as 10^1^ CFU mL^−1^ within 12 h or less, which is significantly less time than the gold standard method (requires several days) for bacterial detection and enumeration. Another method presented by Jin *et al.* [33] was based on CL detection of *Salmonella* via adenosine triphosphate (ATP) quantification on μPAD. *Salmonella* was cultured and then lysed after harvesting by the boiling method. Color change is observed in the μPAD only when ATP is present as an indication of the presence of *Salmonella* in the sample. In the presence of ATP, the HRP-tagged DNA that is initially associated with the ATP aptamer attached to the chemically modified surface of the paper is released and later it allows the catalytic oxidation of 3-amino-9-ethylcarbazole by HRP/H_2_O_2_. The detection limit for *Salmonella* was determined to be 2 × 10^7^ CFU mL^−1^. While no real samples were tested, the developed μPAD could be applied for food and water monitoring. Park *et al.* [46] presented another optical-based technique using a highly angle-dependent and less wavelength-dependent method of detection through a Mie scattering strategy for *Salmonella* Typhimurium. *Salmonella* samples were pre-mixed with anti-*Salmonella* conjugated particles to allow immunoagglutination before loading into the μPAD. At the optimized Mie scatter angle, scatter intensities were analyzed using a smartphone for quantification. An illustration of the μPAD and the smartphone application used for the pathogen quantification are shown in Figure 2a,b, respectively. The detection limit of the smartphone-based μPAD assay was 10^2^ CFU mL^−1^. A one-step multiplexed fluorescence (FL) strategy for detecting pathogens was also developed by Zuo *et al.* [60] using a μPAD that was a hybrid of PDMS and glass. The paper substrate enabled the integration of the fluorescent aptamer-functionalized graphene oxide biosensor on the microfluidic device (Figure 2c). While the aptamer is adsorbed on the surface of the graphene oxide, the FL of the aptamer is quenched. In the presence of the target pathogen, the pathogen induced the liberation of the aptamer from the graphene oxide layer and thereby restored the FL of the aptamer for detection. The detection limits for the simultaneous detection of *S. aureus* and *S. enterica* were 800.0 CFU mL^−1^ and 61.0 CFU mL^−1^, respectively. Other works on *E. coli* detection in water were reported by Burnham *et al.* [57] and Ma *et al.* [30]. Burnham *et al.* specifically demonstrated the use of bacteriophages as capture and sensing elements for the paper-based detection of the pathogen. The method was based on the detection of β-galactosidase released from the pathogenic cells following bacteriophage-mediated lysis. Colorimetric and bioluminescence methods were performed for *E. coli* detection using red-β-d-galactopyranoside chromogenic substrate and Beta-Glo^®^ reagent (Promega Corporation, Madison, WI, USA) to produce the color and bioluminescence, respectively, for measurement with a detection limit of 4 CFU mL^−1^ for both methods. Ma *et al.*, on the other hand, presented a μPAD for the colorimetric determination of *E. coli* using AuNP-labeled detection antibodies via sandwich immunoassay with a silver enhancing step for signal amplification. The detection limit was 57 CFU mL^−1^.

### 3.2. Detection of Pesticides and Herbicides

Pesticides have been used for many years in agriculture and have significantly contributed to maintaining food quality and production. Simultaneously, however, these materials bring harmful effects on human health [61,62]. Wang *et al.* [49] developed a paper-based molecular imprinted polymer-grafted multi-disk micro-disk plate for CL detection of 2,4-dichlorophenoxyacetic acid (2,4-D). The MIP approach was proposed as an alternative to immunoassays, which rely on antibodies and have fundamental drawbacks such as the possible denaturation and instability of the antibodies during manufacture and transport. An indirect competitive assay was made with tobacco peroxidase (TOP)-labeled 2,4-D that was molecularly imprinted on the polymer-grafted device. An enzyme catalyzed CL emission was achieved from the luminol-TOP-H_2_O_2_ CL system with a detection limit of 1.0 pM. A simple paper-based luminol-H_2_O_2_ CL detection of DDV was reported by Liu *et al.* [20]. Paper chromatography was combined in the μPAD CL assay of DDV in fruits and vegetables and the separation was achievable in 12 min utilizing 100 μL of developing reagent. The method was successfully applied to the trace DDV detection on cucumber, tomato and cabbage by a spiking method with a detection limit of 3.6 ng·mL^−1^. Liu *et al.* [21] also presented another MIP-based approach using a paper-based device with a molecularly imprinted polymer for the CL detection of DDV. The detection limit was 0.8 ng·mL^−1^ and the method was successfully applied to cucumber and tomato. A paper-based colorimetric approach has also been demonstrated for the detection of organophosphate and carbamate pesticides. Badawy *et al.* [58] developed a method that was based on the inhibition of acetylcholinesterase (AChE) on the degradation of acetylcholine molecules into choline and acetic acid by organophosphate (methomyl) and carbamate (profenos) pesticides. The degree of inhibition of the AChE indicates the toxicity of the pesticides; this makes the AChE a standard bioevaluator for the presence of organophosphates and carbamates [63]. While the method was not tested on real samples, the method could detect AChE inhibitors within 5 min response time.

**Figure 3:**
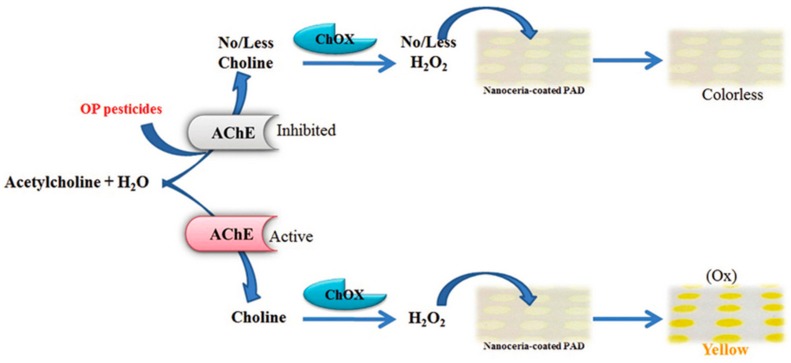
Colorimetric detection of pesticides based on the enzyme inhibition properties of the pesticide on nanoceria substrate. Reprinted with permission from reference [42]. Copyright 2016 The Royal Society of Chemistry.

With the goal to devise portable and easy measuring techniques and considering the increasing use of smartphones, the number of μPAD strategies that incorporate mobile or smartphones for target measurements is increasing. A μPAD sensor and novel smartphone application was developed by Sicard *et al.* [34] for the on-site colorimetric detection of organophosphate pesticides (paraoxon and malathion) based on the inhibition of immobilized AChE by the pesticides. AChE hydrolyzes the colorless indoxyl acetate substrate and converts it to an indigo-colored product in the absence of pesticides. The color intensity is reduced with increasing pesticide concentration owing to inhibition of AChE. The color produced is processed by the image analysis algorithm using a smartphone, allowing real time monitoring and mapping of water quality. The method is capable of detecting pesticide concentration of around 10 nM as evidenced by a color change in the μPAD. Another colorimetric approach was reported by Nouanthavong *et al.* [42] on the use of nanoceria-coated μPAD for colorimetric organophosphate pesticide detection via enzyme-inhibition assay with AChE and choline oxidase. In the presence of the pesticides, AChE activity is inhibited leading to no or less production of H_2_O_2_ and hence less yellow color development of the nanoceria (the color production mechanism is shown in Figure 3). The assay was able to analyze methyl-paraoxon and chlorpyrifos-oxon with detection limits of 18 ng·mL^−1^ and 5.3 ng·mL^−1^, respectively. The method was successfully applied for methyl-paraoxon detection on spiked cabbage and dried green mussel, with ~95% recovery values for both samples.

Another pesticide causing a health concern is pentachlorophenol (PCP) [64,65,66]. PCP is a xenobiotic that accumulates in the body with carcinogenic and acute toxic effects. Sun *et al.* [50] developed a photoelectrochemical (PEC) sensor that utilized the MIP technique on a μPAD to detect PCP. The paper working electrode of the μPAD was covered with a layer of gold nanoparticles (AuNPs) and a layer of polypyrrole (Ppy)-functionalized ZnO nanoparticles. The photoelectrochemical mechanism involves the excitation of electrons from Ppy from its highest occupied molecular orbital to the lowest unoccupied molecular orbital of ZnO after being irradiated with visible light. Since the lowest unoccupied molecular orbital of ZnO and Ppy matched well, the transfer of the excited electrons to ZnO was allowed and the electrons subsequently reached the gold-paper working electrode (Au-PWE) surface, where photocurrent generation efficiency was improved leading to a sharp increase of the photocurrent. However, in the presence of the PCP, the steric hindrance toward the diffusion of the quencher molecules and/or photogenerated holes on the interface of the electrode increased, thereby leading to a decrease in generated photocurrent. The device was capable of measuring PCP down to a limit of 4 pg·mL^−1^.

The only paper-based approach applied to herbicide detection that has utilized FL as a method of detection for methyl viologen is presented by Su *et al.* [67]. The method was based on the integration of CdTe Qdots on the paper device and the CdTe quenching effect in the presence of the target methyl viologen. Presence of a higher methyl viologen concentration in the system gave a darker area on the μPAD as a result of the quenching of the methyl viologen on the CdTe Qdots. The detection limit of the CdTe-paper-based visual sensor was 0.16 μmol·L^−1^.

### 3.3. Detection of Food Additives

In food and beverage industries, wide use is made of food additives such as glucose, fructose and sucrose, which are specifically used as sweeteners, and other food additives, which are used to improve or enhance the flavor or color of the food or beverage. Though most of these food additives are essentially nontoxic, large intakes of them may promote unhealthy nutrition, and some become toxic above a certain amount. Hence, there is a strong demand for fast, highly sensitive and economical methods of analysis that can be provided by the easily accessible and portable point-of-need testing of μPAD technology. Kuek Lawrence *et al.* [51] reported on an amperometric detection of glucose on a screen-printed electrode μPAD. The assay involved the use of ferrocene monocarboxylic acid as a mediator for the catalytic oxidation of glucose on the μPAD by the immobilized glucose oxidase on the paper. The method was successfully applied to glucose detection in commercially marketed carbonated beverages with a limit of 0.18 mM. Adkins *et al.* [35] presented a μPAD that utilized microwire electrodes as an alternative to screen-printed electrodes for the non-enzymatic electrochemical detection of glucose, fructose and sucrose in beverage samples. A copper working electrode was used and the copper electrocatalytically reacted with glucose in the alkaline media, allowing the non-enzymatic electrochemical detection of the carbohydrates. A variety of commercial beverages were tested including Coca-Cola™, Orange Powerade™, Strawberry Lemonade Powerade™, Red Bull™ and Vitamin Water™. The detection limits were 270 nM, 340 nM and 430 nM for glucose, fructose and sucrose, respectively.

Colletes *et al.* [43] presented a study that utilized a paraffin-stamped paper substrate for the detection of glucose in hydrolysis of liquors (detection limit 2.77 mmol·L^−1^) by paper spray mass spectrometry (PS-MS). PS-MS is a fast, precise, accurate and cost-effective ionization method introduced by Crooks and co-workers in 2010 that provides complex analyses in a simple and economical way by mass spectrometry [68]. Although the paraffin-stamped paper substrate is not a μPAD *per se,* Colletes *et al.* explained the potential of the paper substrate for the combination of a microfluidic paper-based analytical device with mass spectrometry that used paper spray as the ionization method.

**Figure 4:**
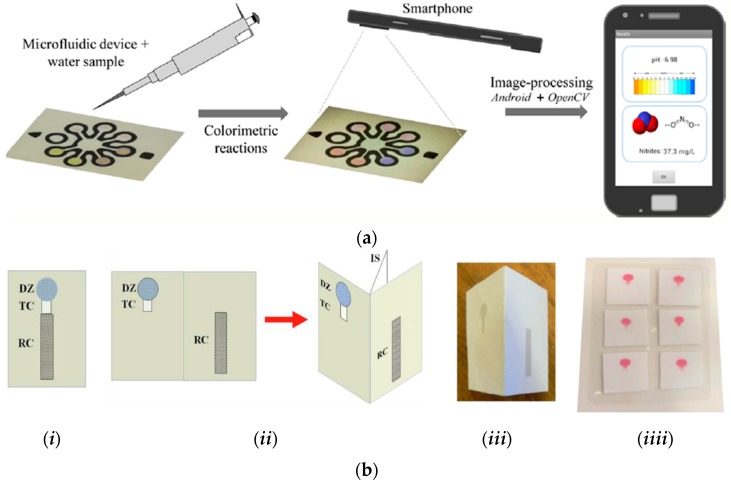
(**a**) Griess-color reaction assay-based detection methods for nitrite using a smartphone for image processing. Reprinted with permission from reference [45]. Copyright 2014 American Chemical Society. (**b**) Griess-color reaction assay-based detection methods for nitrite and nitrate using 2D (*i*) and 3D (*ii–iv*) μPADs. Reprinted with permission from reference [53]. Copyright 2014 American Chemical Society.

Nitrites are food additives used to prevent the growth of microorganisms as well as to inhibit lipid oxidation that causes rancidity [69]. Nitrite monitoring in food and water is essential due to the ability of nitrite to readily react with secondary and tertiary amines and produce carcinogenic nitrosamine compounds [70]. Several works on nitrite detection have involved the use of the Griess-color reaction mechanism to visually detect the presence of nitrite in food. For instance, He *et al.* [52] described a μPAD using the Griess-color nitrite assay, where, upon reaction of nitrite with the Griess reagent in the μPAD, a color developed with intensities depending on the amount of nitrite in the sample. Image processing was done for quantification showing a dynamic range of 0.156–2.50 mM, and a successful application to nitrite detection in red cubilose (a traditional nutritious food and medicine in China) was achieved. Other works presented by Lopez-Ruiz *et al.* [45], Cardoso *et al.* [44] and Jayawardane *et al.* [53] similarly focused on the colorimetric detection of nitrite in water and food using the Griess method in μPADs. Lopez-Ruiz *et al.* presented a strategy using a mobile phone with a customized algorithm for image analysis and detection. As depicted in Figure 4a, the method allowed a multidetection of the μPAD sensing areas specific for pH detection simultaneously with nitrite detection in water samples. The strategy involved capturing the μPAD image upon sample detection with the smartphone camera, and processing of the image in order to extract the colorimetric information for measurement, wherein, hue (H) and saturation (S) of the HSV color space were used for the determination of pH and nitrite concentration, respectively. The colorimetric assay for pH determination was based on the use of two pH indicators, phenol red and chlorophenol red. A color transition of chlorophenol red from yellow to purple indicated a pH from 4 to 6, while a color transition of phenol red from yellow to pink indicated a pH from 6 to 9. The nitrite assay, on the other hand, involved a Griess-color reaction in which the color formation was quantitatively interpreted showing a detection limit of 0.52 mg·L^−1^. Cardoso *et al.* similarly reported a μPAD strategy for nitrite detection in ham, sausage and the preservative water from a bottle of Vienna sausage using the Griess-color assay with a detection limit of 5.6 μM. The colorimetric analysis was performed by first taking the image of the detection device using a scanner, and later processing the magenta scale of the image after conversion to the CMYK using Corel Photo-Paint™ software. Finally, Jayawardane *et al.* presented their work for nitrite and nitrate determination in different water samples using two μPADs, each specific for nitrate and nitrite, respectively. The image of the 2D and 3D μPADs used for detection are shown in Figure 4b. The nitrite detection simply employed the Griess method for colorimetric measurements after image scanning and processing using ImageJ software. In the nitrate detection however, a conversion of the colorimetrically undetected species was first performed to the colorimetrically detected nitrite using a Zn reduction channel incorporated in the μPAD for nitrate detection. After conversion, the Griess method was employed and image quantification was performed. The method was successfully applied to actual analysis of different water samples (tap water, mineral water, and pond water) with detection limits of 1.0 μM and 19 μM for nitrite and nitrate, respectively.

The addition of colorants to food has become a normal practice to enhance or change food color and make it more attractive to consumers. However, most of these colorants are potentially harmful to human health especially after excessive consumption. One μPAD design that has been developed for detecting colorants was presented in the work of Zhu *et al.* [22] where a poly(sodium 4-styrenesulfonate)-functionalized paper substrate was used for the rapid separation, preconcentration and detection of colorants in drinks with complex components via a surface-enhanced Raman spectroscopy (SERS) method. Sunset yellow and lemon yellow were both detected in grape juice and orange juice with detection limits of 10^−5^ M and 10^−4^ M, respectively.

### 3.4. Detection of Heavy Metals

**Figure 5:**
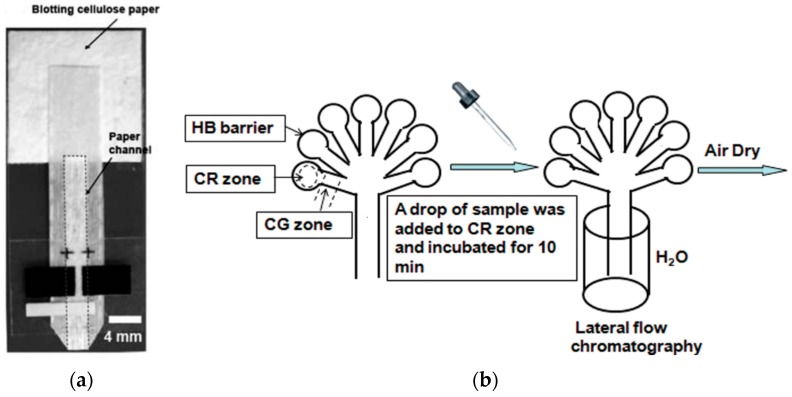
Detection methods for metals. (**a**) Electrochemical device for SWASV analysis of lead in water with screen-printed carbon working and counter electrodes and Ag/AgCl pseudo-reference electrode. Reprinted with permission from reference [47]. Copyright 2009 The Royal Society of Chemistry. (**b**) Multiplexed colorimetric detection of metals based on B-GAL and CPRG interaction in the presence of Hg^2+^, Cu^2+^, Cr^6+^ and Ni^2+^ mixture. Reprinted with permission from reference [31]. Copyright 2011 American Chemical Society.

Several μPADs have been developed for the detection of heavy metals in both food and water. The most common methods of detection integrated with the μPADs were colorimetric-based using silver or gold nanoparticles and nanoplates, but electrochemical and FL based methods were used as well. Nie *et al.* [47] developed a μPAD for the versatile and quantitative electrochemical detection of biological and inorganic analytes in aqueous solutions. Specifically, for water analysis, lead was investigated via square wave anodic stripping voltammetry using a μPAD with screen-printed electrodes as shown in Figure 5a. The measurements relied on the simultaneous plating of bismuth and lead onto the screen-printed carbon electrodes of the μPAD, which formed alloys, followed by anodic stripping of the metals from the electrode. The method showed a detection limit of 1.0 ppb in water medium. Similarly, Shi *et al.* [54] developed an electrochemical μPAD for Pb(II) and Cd(II) detection based on square wave anodic stripping voltammetry (SWASV) relying on *in situ* plating of bismuth film. The method was capable of detecting lead and cadmium ions simultaneously in carbonated electrolyte drink (salty soda water as described by the authors) samples with detection limits of 2.0 ppb and 2.3 ppb for Pb(II) and Cd(II), respectively.

Using silver nanoparticles (AgNP) self-assembled with aminothiol compounds on μPADs, Ratnarathorn *et al.* [25] reported on the colorimetric detection of copper in drinking water samples. In the presence of Cu^2+^, the modified AgNP solution changed from yellow to orange and then green-brown due to nanoparticle aggregation. The method was tested on tap water and pond water samples with a detection limit of 7.8 nM or 0.5 μg·L^−1^. Two other applications of μPAD with colorimetric detection for Cu(II) were reported by Jayawardane *et al.* [55] and Chaiyo *et al.* [36]. In the former work, a polymer inclusion membrane (PIM) containing the chromophore (1-(2′-pyridylazo)-2-naphthol (PAN)) reactive to Cu(II) was incorporated in the μPAD and was used as the sensing element selective to the metal ion. The original yellow color of the membrane changed to red/purple as the Cu(II) formed a complex with PAN. The device was applied to Cu(II) determination in hot tap water samples with a detection limit of 0.6 mg·L^−1^. The latter work by Chaiyo *et al.* on the other hand used silver nanoplates (AgNPls) modified with hexadecyltrimethyl-ammonium bromide (CTAB) for the colorimetric detection of Cu(II) based on the catalytic etching of the AgNPls with thiosulfate (S_2_O_3_^2−^). The violet-red S_2_O_3_^2−^/CTAB/AgNPl on the detection zone lost its color with increasing Cu^2+^ concentration. The method was applied for determination of Cu^2+^ in drinking water, ground water, tomato and rice with a detection limit of 1.0 ng·mL^−1^ by visual detection. Nath *et al.* [23] presented a sensing system that could detect As^3+^ ions using gold nanoparticles chemically conjugated with thioctic acid (TA) and thioguanine (TG) molecules on paper. During detection, a visible bluish-black color appeared on the paper due to nanoparticle aggregation through transverse diffusive mixing of the Au–TA–TG with As^3+^ ions. While no real water sample testing was performed, the detection limit (1.0 ppb) was lower than the reference standard of World Health Organization (WHO) for arsenic in drinking water, hence there would be method applicability to real water sample analysis. Another work presented by the same group used a similar approach for the detection of Pb^2+^ and Cu^2+^ using AuNP that was chemically conjugated with TA and dansylhydrazine [24]. The detection limit was ≤0.0 ppb for both metal ions. Apilux *et al.* [41] developed a colorimetric method using AgNPls for the detection of Hg(II) ion levels. A change in color from pinkish violet to pinkish yellow occurred with the Hg(II) ion detection, a phenomenon that can be attributed to a change in the surface plasmon resonance of the AgNPls, which is related to the AgNPl apparent color. At Hg(II) concentration levels above 25 ppm, the color of the AgNPls fades as observed by the naked eye. With digital imaging and software processing though, the quantitative capability of the system was improved and showed a detection limit of 0.12 ppm with successful applications to real sample analysis of drinking water and tap water. Another method via FL detection for the determination of Hg(II), Ag(I) and neomycin (NEO) for food analysis was presented by Zhang *et al.* [37]. The method used a Cy5-labeled single-stranded DNA (ssDNA)-functionalized graphene oxide (GO) sensor that generated FL in the presence of the target analytes, otherwise, the Cy5 was quenched while adsorbed on the GO surface. The detection limits were 121 nM, 47 nM and 153 nM for Hg(II), Ag(I) and NEO, respectively.

Hossain *et al.* [31] presented a multiplexed μPAD that is capable of detecting heavy metals simultaneously in a single μPAD. As shown in Figure 5b, the μPAD is composed of seven reaction zones, two of which are for control experiments, one for testing the mixture of metal ions via β-galactosidase (B-GAL) assay, and four using colorimetric reagents specific for Hg(II), Cu(II), Cr(VI) and Ni(II), respectively. In the B-GAL assay, the chromogenic substrate, chlorophenol red β-galactopyranoside (CPRG), which is printed on a region upstream to the B-GAL zone, is transported into the detection zone by the sample solution through capillary action and it is hydrolyzed by the B-GAL enzyme to form the red-magenta product. In the presence of the metal ions, the red-magenta color produced upon CPRG hydrolysis is lost to a degree dependent on the concentration of the metal ions in the sample. For the assays specific for each metal ion, color appearance is observed in the presence of each metal ion on their respective detection zones, while the absence of any of the metal ions results in no color change on the respective zones. The detection limit of the device is ~0.5–1.0 ppm. Li *et al.* [28] demonstrated the use of a μPAD that enables easy detection of trace metals via text-reporting of results. Using the color-generating periodic table symbols of the specific trace metals fabricated on the μPAD as markers, even nonprofessional users can carry out handy detection and monitoring. The Cu(II) assay was based on the formation of an orange to brown complex by bathocuproine as the indicator with Cu(II). For the Cr(VI) assay, a magenta to purple complex formed in the presence of the metal ion with the indicator 1,5-diphenylcarbazide in acidic medium, while for the Ni(II) assay, a stable pink-magenta colored complex formed between dimethylglyoxime and Ni(II). The device was capable of colorimetric detection of Cu(II), Cr(VI) and Ni(II) in tap water with concentrations of ≥0.8 mg·L^−1^, >0.5 mg·L^−1^ and ≥0.5 mg·L^−1^, respectively. Finally, for μPAD detection of heavy metals, a colorimetric approach for image processing and quantification based on an iron-phenanthroline (Fe-phen) assay that has colored response with increasing concentration of iron was incorporated for the investigation of iron in water samples by Asano *et al.* [48]. The developed method allowed a direct analysis of tap and river water samples without pretreatment with a detection limit of 3.96 μM.

### 3.5. Detection of Other Food and Water Contaminants

**Figure 6:**
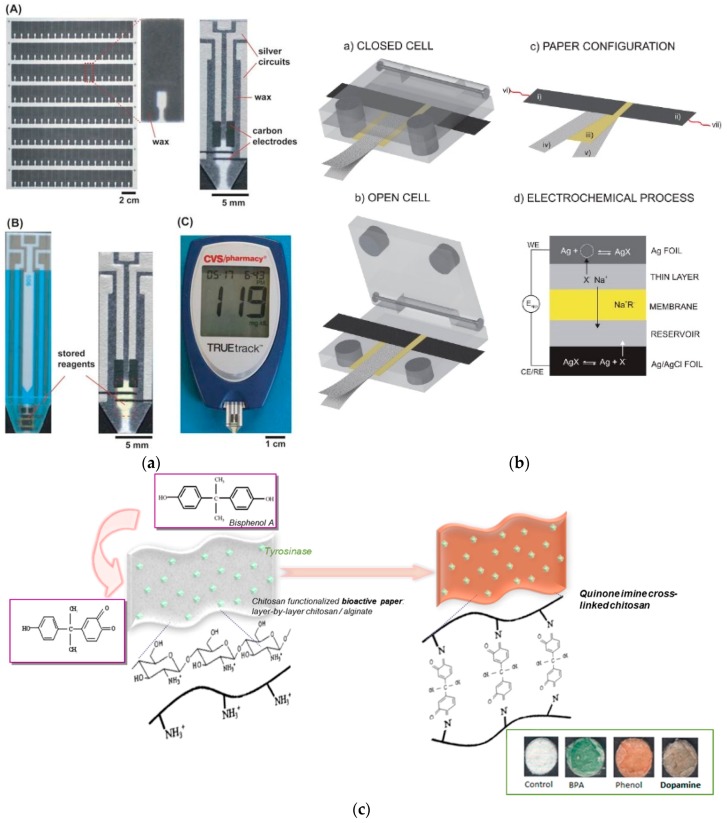
Detection methods for other food and water contaminants. (**a**) Components of the electrochemical detection system for ethanol using a glucometer as a readout device. Reprinted with permission from reference [38]. Copyright 2010 The Royal Society of Chemistry. (**b**) The configuration of the electrochemical cell for the analysis of halides utilizing silver components as electrodes on paper-assisted electrochemical detection. Reprinted with permission from reference [56]. Copyright 2015 American Chemical Society. (**c**) A representative paper-based colorimetric bioassay of BSA based on the enzymatically generated quinone from tyrosinase and chitosan interaction in the presence of the phenolic compound. Reprinted with permission from ref [59]. Copyright 2012 American Chemical Society.

Several methods have also been demonstrated for detecting other food and water contaminants using μPAD technology. Nie *et al.* [38] presented an electrochemical technique for ethanol detection in water for possible food quality control purposes. Electrochemical μPADs and a glucometer (Figure 6a) were used to amperometrically measure ethanol (LOD 0.1 mM) using ferricyanide as an electron-transfer mediator and alcohol dehydrogenase/β-NAD^+^ as detecting components in the device. An electrochemical μPAD for halide detection in food supplement and water samples via cyclic voltammetry was also developed by Cuartero *et al.* [56]. The device utilizes silver elements as working and counter/reference electrodes as illustrated in Figure 6b. The oxidation of the silver foil working electrode is induced by an anodic potential scan resulting in a current that is related to the plating rate of the target halides in the sample as silver halides precipitate. This process is complemented by the reduction of the silver/silver halide element in the reference/counter electrode upon ion exchange movement of the Na^+^ ion (halide counterion) through the permselective membrane to maintain the neutrality of charges in each paper compartment, and that leads to the release of halide ions into the solution. The two silver elements are regenerated to their previous states through the application of a backward potential sweep after the forward scan. The device was found capable of detecting bromide, iodide and chloride mixtures in food supplement, seawater, mineral water, tap water and river water samples with a detection limit of around 10^−5^ M of halide mixtures. Myers *et al.* [39] developed a multiplexed μPAD (called a saltPAD) that is capable of making an iodometric titration in a single printed card. Multiple reagents are stored on every compartment of each detection zone of the saltPAD and they are allowed to recombine and undergo surface-tension-enabled mixing upon introduction of the iodized salt sample solution for determination. During the iodometric titration process, triiodide is formed as excess iodide that reacts with iodate in the presence of acid. The triiodide is then titrated with thiosulfate that was previously stored in the saltPAD. Using starch as an indicator, the detection zone produces a blue color if the amount of triiodide exceeds the reducing capacity of the thiosulfate. The indicator remains uncolored if the amount of triiodide is smaller than the reducing capacity of the thiosulfate. The detection limit of the device expressed as mg iodine/kg salt was 0.8 ppm.

Cyanobacteria in drinking water pose a great threat to public health due to the cyanotoxins produced and released into water supplies. The most toxic of the cyanotoxins is microcystin-LR (MC-LR) [71,72]. Ge *et al.* [40] focused on the development of a method that specifically detects MC-LR in water using a gold-paper working electrode (Au-PWE) for electrochemical immunoassay. Differential pulse voltammetric measurements were performed by monitoring the oxidation process of thionine in the system for the quantification of MC-LR under the catalysis of HRP and peroxidase mimetics (Fe_3_O_4_). The sandwich immunoreaction produced a current proportional to the logarithm of MC-LR and gave a detection limit of 0.004 μg·mL^−1^. Phenolic compounds are generally produced as byproducts from industrial processes that present health risks to humans after consumption of contaminated food and water. For detection of phenolic compounds, Alkasir *et al.* [59] developed a paper sensor that produces different color responses for phenol (reddish-brown), bisphenol A (blue-green), dopamine (dark-brown), cathecol (orange), and m-cresol (orange) and p-cresol (orange) resulting from the specific binding of enzymatically generated quinone to chitosan immobilized in multiple layers on the paper. Figure 6c illustrates an example of the layer-by-layer paper-based bioassay for bisphenol A. The paper sensor was successfully applied to the analysis of tap and river water samples with a detection limit of 0.86 (±0.102) μg·L^−1^ for each of the phenolic compounds.

Finally, the only μPAD detection strategy based on electrochemiluminescence (ECL) detection for the specific analysis of food has been reported by Mani *et al.* [29]. The work described a device that specifically measures the genotoxic activity of a certain compound (benzo[a]-pyrene (B[a]P)) whose metabolite reacts with DNA and the responses are measured via ECL detection. The measurement essentially involves two steps, the first of which involves the conversion of the test compound B[a]P to a metabolite by a microsomal enzyme from rat liver microsomes. The second step is a DNA damage detection that involves the liberation of ECL light upon oxidation of the guanine in the damaged DNA by the (bis-2,2′-bipyridyl) ruthenium polyvinylpyridine ([Ru(bpy)_2_(PVP)_10_]^2+^ or RuPVP) polymer of the electrochemical device. The technique was specifically tested on grilled chicken, and the detection limit was ~150 nM.

## 4. Conclusions and Future Directions

A review of microfluidic paper-based devices for food and water analysis has been presented. Table A1 (Appendix A) summarizes uses of microfluidic paper-based devices for detection of different pathogens, additives and contaminants in food and water that have been reported to date. μPADs in food and water safety and analysis represent a burgeoning technology that provides fast, economic, easy-to-use advantages and is highly applicable for point-of-need testing especially in resource limited environments. While the field of microfluidic paper-based sensors has expanded rapidly, food and water safety remains an area with many issues still to be addressed. One specific challenge in food analysis for example is the method of handling and pretreatment of the samples before μPAD detection. While fluid samples such as water and beverage usually do not require any pretreatment to the sample before introducing into the device for μPAD detection [22,28,38,48,49,51,53,54,55,56,59], food specimens could be in solid form, and therefore, a suitable pretreatment step is necessary for target sample collection before introducing into the μPAD for detection. In treating fruits, vegetables and meat samples for instance, most groups employ an extraction method to collect the target of interest [29,42], although an elution process [20,21], or boiling method [44], with the use of distilled water, followed by filtration are simple steps that are possibly performed to collect the target for μPAD detection. For pathogen collection, the swab sampling technique has also been performed which requires a significantly reduced enrichment times compared to the gold standard culture method before sample introduction and colorimetric paper-based detection [32]. While successful, the enzymatic assay systems point to the potential for exploring the use of specific inducers to enhance enzyme production as well as using selective enrichment media to inhibit the growth of competing microorganisms. Despite the current limitations on selectivity and sensitivity using paper as substrates for detection, the ability of μPADs to detect specific targets such as pathogenic bacteria, food additives and contaminants has been demonstrated in real food and water samples at levels that are vital to the safety and health of both animals and humans, therefore demonstrating its significant impact to the community for food and water safety and quality monitoring. Based on the number of references reporting the development of μPADs specifically directed to food and water safety and quality monitoring in the last six years, μPAD technology is still in its early stage and there are wide opportunities for developments and applications. Particularly exciting is the potential for application of μPADs for regular monitoring of food crops and drinking water sources, where, contamination is a risk from mining and industrial processes, and analytical measurements have traditionally been a cost limiting factor. From the detection of foodborne and waterborne infectious pathogens to different organic and inorganic analytes in general, μPADs offer the means to detect different targets using an inexpensive material like paper as their main substrate for qualitative as well as quantitative on-site food and water monitoring.

## Abbreviations

The following abbreviations are used in this manuscript:

2,4-D	2,4-dichlorophenoxyacetic acid
Ach	acetylcholinesterase
AgNP	silver nanoparticle
AgNPl	silver nanoplate
ATP	adenosine triphosphate
B[a]P	benzo[a]pyrene
B-GAL	β-galactosidase
BPA	bisphenol A
CFU	colony-forming unit
CL	chemiluminescence
CMYK	cyan-magenta-yellow-key
CPRG	chlorophenol red β-galactopyranoside
DDV	dichlorvos
*E. coli*	*Escherichia coli*
ECL	electrochemiluminescence
FL	fluorescence
GO	graphene oxide
HRP	horseradish peroxidase
*L. monocytogenes*	*Listeria monocytogenes*
LOD	limit of detection
MCE	mixed cellulose esters
MC-LR	microcystin-LR
MIP	molecularly imprinted polymer
NEO	neomycin
PCP	pentachlorophenol
PDMS	poly(dimethylsiloxane)
PEC	photoelectrochemical detection
PIM	polymer inclusion membrane
Ppy	polypyrrole
PS-MS	paper spray mass spectrometry
Qdots	quantum dots
*S. aureus*	*Staphylococcus aureus*
*S. enterica*	*Salmonella enterica*
*S.* Typhimurium	*Salmonella* Typhimurium
SERS	surface-enhanced Raman spectroscopy
SWASV	square wave anodic stripping voltammetry
μPAD	microfluidic paper-based analytical device

## Appendix A

**Table A1:** Summary of foodborne pathogens, toxins, pesticides and insecticides, heavy metals and food additives for food and water analyses on paper-based platforms.

Target	μPAD Wall Fabrication Method	Paper Substrate	Detection Method	Linear Detection Range	LOD	Real Sample Application	Reference
***Pathogens***							
*E. coli* O157:H7, *Salmonella* Typhimurium, *L. monocytogenes*	Wax printing	Whatman No. 1 filter paper	Colorimetric	-	10^6^ CFU mL^−1^, 10^4^ CFU mL^−1^, 10^8^ CFU mL^−1^	Bologna	[ 32]
*Salmonella*	Wax printing	Whatman No. 1 chromatography paper	CL	-	2.6 × 10^7^ CFU mL^−1^	-	[ 33]
*S.* Typhimurium	Photolithography	Chromatography paper	Optical (Mie scattering)	10^2^–10^5^ CFU mL^−1^*	10^2^ CFU mL^−1^	-	[ 46]
*S. aureus, S. enterica*	Cutting by punching (PDMS/paper/glass hybrid)	Whatman chromatography paper	FL	10^4^–10^6^ CFU mL^−1^, 42.2–675.0 CFU mL^−1^	800.0 CFU mL^−1^, 61.0 CFU mL^−1^	-	[ 60]
*E. coli*	-	Millipore MCE membrane filter	Colorimetric and bioluminescence	-	4 CFU mL^−1^	-	[ 57]
*E. coli*	Wax pencil drawing and PDMS screen printing	Whatman No. 1 filter paper	Colorimetric	-	57 CFU mL^−1^	Drinking water	[ 30]
***Pesticides and Herbicides***							
2,4-D	-	Whatman No. 1 chromatography paper	CL	-	1.0 pM	Tap water, lake water	[ 49]
Paraoxon, Malathion	Wax printing	Whatman No. 1 filter paper	Colorimetric	1 × 10^−8^–ca. 1 × 10^−6^ M	10 nM	-	[ 34]
Methyl-paraoxon, Chlorpyrifos-oxon	Polymer screen-printing	Whatman No. 4 filter paper	Colorimetric	0–0.1 μg·mL^−1^, 0–60 ng·mL^−1^	18 ng·mL^−1^, 5.3 ng·mL^−1^	For methyl-paraoxon: cabbage, dried green mussel	[ 42]
Dichlorvos	Cutting	Whatman 3MM Chr chromatography paper	CL	10 ng·mL^−1^–1.0 μg·mL^−1^	3.6 ng·mL^−1^	Cucumber, tomato, cabbage	[ 20]
Dichlorvos	Cutting	Whatman 3MM Chr chromatography paper	CL	3.0 ng·mL^−1^–1.0 μg·mL^−1^	0.8 ng·mL^−1^	Cabbage, tomato	[ 21]
Methomyl, Profenofos	Cutting	Canson paper	Colorimetric	-	6.16 × 10^−4^ mM, 0.27 mM	-	[ 58]
PCP	Wax screen-printing	Whatman No. 1 chromatography paper	PEC	0.01–100 ng·mL^−1^	4 pg·mL^−1^	-	[ 50]
Methyl viologen (paraquat)	Cutting	Whatman filter paper	FL	0.39 μmol·L^−1^–3.89 μmol·L^−1^	0.16 μmol·L^−1^	-	[ 67]
***Food Additives***							
Glucose	Cutting by punching	Whatman No. 1 filter paper	Electrochemical	1–5 mM	0.18 mM	Commercial soda beverages	[ 51]
Glucose, Fructose, Sucrose	Wax printing	Whatman No. 1 filter paper	Electrochemical	-	270 nM, 340 nM, 430 nM	Coca-Cola™, Orange Powerade™, Strawberry Lemonade Powerade™, Red Bull™, Vitamin Water™	[ 35]
Glucose	Paraffin stamping	Whatman grade 1 paper	PS-MS	1–500 μmol·L^−1^	2.77 μmol·L^−1^	Liquors	[ 43]
Sunset yellow, Lemon yellow	Cutting	Filter paper	SERS	-	10^−5^ M, 10^−4^ M	Grape juice, orange juice	[ 22]
Nitrite	Paraffin stamping	JProLab JP 40 filter paper	Colorimetric	0–100 μM	5.6 μM	Ham, sausage, preservative water	[ 44]
Nitrite	Alkylsilane assembling and UV-lithography	Whatman No. 1 filter paper	Colorimetric	0.156–2.50 mM	-	Processed red cubilose	[ 52]
Nitrite	Indelible ink contact stamping	Whatman No. 1 filter paper	Colorimetric	-	0.52 mg·L^−1^	-	[ 45]
Nitrite, Nitrate	Inkjet printing	Whatman No. 1 and No.4 filter papers	Colorimetric	10–150 μM, 50–1000 μM	1.0 μM, 19 μM	Tap water, mineral water, pond water	[ 53]
***Metals***							
Pb(II)	Photolithography	Whatman No. 1 chromatography paper	Electrochemical	0–100 ppb	1.0 ppb	-	[ 47]
Hg(II), Cu(II), Cr(VI), Ni(II)	Wax printing	Whatman No. 1 paper	Colorimetric	-	~0.5–1 ppm	-	[ 31]
Pb(II), Cd(II)	Cutting	Whatman No. 1 filter paper	Electrochemical	10–100 ppb	2.0 ppb, 2.3 ppb	Carbonated electrolyte drinks	[ 54]
As(III)	Cutting	Whatman filter paper	Colorimetric	-	1.0 ppb	-	[ 23]
Pb(II), Cu(II)	Cutting	Whatman filter paper	Colorimetric	-	≤10.0 ppb for both	-	[ 24]
Cu(II)	Cutting	Whatman No. 1 filter paper	Colorimetric	7.8–62.8 μM	7.8 nM or 0.5 μg·L^−1^	Drinking water	[ 25]
Cu(II)	Wax printing	Whatman No. 1 filter paper	Colorimetric	0.5–200 ng·mL^−1^	0.3 ng·mL^−1^	Drinking water, ground water, tomato, rice	[ 36]
Cu(II)	Inkjet printing	Whatman No. 4 filter paper	Colorimetric	0.1–30.0 mg·L^−1^	0.6 mg·L^−1^	Hot tap water	[ 55]
Hg(II)	Wax screen printing	Whatman No. 1 filter paper	Colorimetric	5–75 ppm	0.12 ppm	Commercial bottled drinking water, tap water	[ 41]
Hg(II), Ag(I), NEO	Wax printing	Whatman No. 1 chromatography paper	FL	0–3 μM, 0–1.75 μM, 0–2 μM	121 nM, 47 nM, 153 nM	-	[ 37]
Cu(II), Cr(VI), Ni(II)	Wax patterning	Whatman No. 1 chromatography paper	Colorimetric	-	≥0.8 mg·L^−1^, >0.5 mg·L^−1^, ≥0.5 mg·L^−1^	Tap water	[ 28]
Fe	Photolithography	Advantec No. 51B chromatography paper	Colorimetric	8.9–89 μM	3.96 μM	Tap water, river water	[ 48]
***Others***							
Ethanol	Wax printing	Whatman No. 1 chromatography paper	Electrochemical	0.1–3 mM	0.1 mM	Water	[ 38]
Phenol, Bisphenol A, Dopamine, Catechol, m-Cresol p-Cresol	Cutting by hole punching	Fisherbrand P5 filter paper	Colorimetric	1–400 μg·L^−1^, 1–200 μg·L^−1^, 1–300 μg·L^−1^, 1–300 μg·L^−1^, 1–500 μg·L^−1^, 1–200 μg·L^−1^	0.86 (±0.102) μg·L^−1^ for each of the phenolic compounds	Tap water, river water	[ 59]
Bromide, Iodide, Chloride	-	Whatman RC60 regenerated cellulose membrane filter	Electrochemical	10^−4.8^–0.1 M for bromide and iodide, 10^−4.8^–0.6 M for chloride	10^−5^ M	Food supplement, seawater, mineral water, tap water, river water	[ 56]
Iodate	Wax printing	Ahlstrom 319 paper	Colorimetric	0.8–15 ppm iodine atoms from iodate	0.8 ppm iodine atoms	Iodized salt	[ 39]
MC-LR	Wax printing	Whatman No. 1 chromatography paper	Electrochemical	0.01–200 μg·mL^−1^	0.004 μg·mL^−1^	-	[ 40]
B[a]P	Wax patterning and screen printing	Whatman No. 1 filter paper	ECL	0.15–12.5 μM	~150 nM	Chicken skin	[ 29]
